# A Perspective on Re-Detectable Positive SARS-CoV-2 Nucleic Acid Results in Recovered COVID-19 Patients

**DOI:** 10.1017/dmp.2020.392

**Published:** 2020-10-22

**Authors:** Yanfei He, Yu-Chao Dong

**Affiliations:** Department of Cadre Health Care, Sixth Medical Center, Chinese PLA General Hospital, Beijing, China; Department of Respiratory and Critical Care Medicine, Shanghai Hospital, Naval Medical University, Shanghai, China

**Keywords:** COVID-19, re-detectable positive, SARS-CoV-2

## Abstract

**Objectives:**

There have been reports on re-detectable positive nucleic acid tests for severe acute respiratory syndrome coronavirus 2 (SARS-CoV-2) in recovered coronavirus disease (COVID-19) patients. In this study, we look at the clinical characteristics, possible causes, pathogenesis, and infectivity of re-detectable positive patients and provide up-to-date information to public health policy planners and clinicians.

**Methods:**

By consulting the latest research data and related progress data of re-detectable positive patients, this study addresses the implications that this special group brings to clinical work and disease prevention and control.

**Results:**

We discuss in detail the phenomenon of re-detectable positive nucleic acid tests for recovered patients. There are many possible causes of a re-detectable positive, but there is no 1 factor that can fully explain this phenomenon.

**Conclusions:**

It can’t be completely ruled out that the re-detectable positive patients are infectious. We should be alert to these re-detectable positive patients becoming chronic virus carriers, and virus serological IgM and IgG antibody tests should be added before patient discharge. It is urgent to find a more powerful evidence-based and virological basis for the integrity of viral ribonucleic acid and the variation of viral virulence with time through cell experiments in vitro and animal experiments in vivo.

Since December 2019, many cases of the novel coronavirus (2019-nCoV)-infected pneumonia have been discovered in Wuhan, China,^1^ and such cases have spread nationwide quickly.^2^ It was named *coronavirus disease* (*COVID-19*) by the World Health Organization on February 11, 2020. As of May 5, 2020, a total of 3 517 345 cases have been confirmed worldwide with 243 401 deaths.^3^ It is known that COVID-19 is caused by severe acute respiratory syndrome coronavirus 2 (SARS-CoV-2) or 2019-nCoV.^4,5^ It is transmitted mainly through close contact and droplets, and there have also been reports of possible transmission by aerosol and fecal-oral routes.^6,7^ The main symptoms are fever, fatigue, and a dry cough. The diagnosis is mainly based on clinical symptoms, epidemiological history, chest imaging findings, laboratory examination, and the ribonucleic acid (RNA) test of SARS-CoV-2.^8^ Recently, an increasing number of patients with COVID-19 have been discharged from the hospital and have received regular follow-up and observation. A re-detectable positive of the SARS-CoV-2 RNA tests in some recovered patients has been reported,^9,10^ and this phenomenon is receiving more and more attention.

## WHO ARE THE RE-DETECTABLE POSITIVE PATIENTS?

The re-detectable positives refer to patients who meet the discharge criteria, have no respiratory symptoms, chest CT images show obvious absorption of inflammation, respiratory tract samples are re-examined after discharge, and the SARS-CoV-2 RNA test is positive again. Of course, there are also reports that asymptomatic carriers can be a re-detectable positive.^11^

## WHAT ARE THE CLINICAL CHARACTERISTICS OF RE-DETECTABLE POSITIVE PATIENTS?

Studies have shown that re-detectable positive patients have 2 characteristics in common.^12^ First, the original clinical symptoms are light. Almost all re-detectable positive cases are concentrated in patients with light symptoms as compared to severe cases. After readmission to the hospital for isolation treatment, these re-detectable positive patients had no evidence of disease progression or aggravation, signs and symptoms were further improved, and reexamination of chest CTs showed that there were no inflammatory lesions in the lungs or the original residual inflammatory lesions continued to decrease, and there were very few cases of deterioration. Second, these patients are very young. Children less than 14 years old accounted for 35.0% of the re-detectable positive patients of the same age, and patients between the ages of 14 and 60 years accounted for 16.0% of the re-detectable positive patients of the same age.

## ARE THE RE-DETECTABLE POSITIVE PATIENTS INFECTIOUS?

Based on current detection techniques, SARS-CoV-2 RNA test results are qualitative rather than quantitative, so a positive result of reverse transcription quantitative polymerase chain reaction (RT-qPCR) does not necessarily mean the person is still infectious, as RNA may come from an inactive or lethal virus.^13^ Although there is no evidence of the re-detectable positive patients infecting others,^14^ since it is not clear whether the positive results of PCR are caused by active viral particles or non-infectious viral gene fragments, the risk of infection of the re-detectable positive patients cannot be completely ruled out.

## WHAT ARE THE REASONS FOR THE RE-DETECTABLE POSITIVE?

The specific pathological mechanism of a re-detectable positive is still unclear. There are many possible causes of a re-detectable positive.

The first possible reason is reinfection. Feng XH et al.^15^ studied 5 re-detectable positive patients isolated in a single room, and none of them came into contact with a new source of infection during isolation, which confirmed that the cause of the nucleic acid re-detectable positive was not likely caused by reinfection after discharge. But, just recently, Kelvin K-W et al.^16^ reported a 33-year-old male patient living in Hong Kong who developed his second infection 142 days after his first infection with SARS-CoV-2. The whole-genome analysis showed that the strain of the second infection was completely different from that of the first infection, and his epidemiological history, clinical manifestations, and serological data supported the argument of reinfection. Bao et al.’s research found that rhesus macaques infected with SARS-CoV-2 will not be infected with the same virus strain after recovery, indicating that neutralizing antibodies against SARS-CoV-2 might protect rhesus macaques that have undergone an initial infection from reinfection during early recovery days.^17^ However, researchers also believe that there are great differences between humans and rhesus macaques, and more research is needed on whether people infected with SARS-CoV-2 can prevent reinfection. The cases of reinfection in Hong Kong, China, and the United States^18^ support a similar view that the level of immunity produced by the first infection of COVID-19 may not have a 100% protective effect on everyone. Whether these reinfected cases have their extremely special situation, and how common the phenomenon of reinfection is after recovery, we do not know at present. It is necessary to have a better understanding of the pattern of SARS-CoV-2 through the study of more recovered patients and the tracking of virus gene sequence changes.

The second possible reason is related to sampling. Sampling materials (swabs and preservation solution), sampling quantity, sampling time, and the conduct of samplers can affect the nucleic acid detection results.^13,19^ It is also reported that the quality, stability, and reliability of the nucleic acid detection kit may also lead to false-negative results of nucleic acid amplification at discharge and positive at retest.^20^ Luo et al. reported that 8 of the 20 re-detectable positive patients were persistently positive for SARS-CoV-2 RNA in Guangzhou, China. These 20 patients were regularly sampled and tested for SARS-CoV-2 RNA by professionals from the Guangzhou Centers for Disease Control and Prevention, and all the sampling personnel had received strict training. All SARS-CoV-2 RNA detection reagents met national standards, and for the positive results of nucleic acid detection, 2 kinds of kits were used to confirm the results.^21^ This indicates that the difference in the quality of nucleic acid detection kits cannot fully explain the re-detectable positive phenomenon.

Besides, the location and quality of samples are also important factors for the results of viral RNA detection.^22^ It has been reported that the viral load of nasal swabs is higher than that of pharyngeal swabs,^23^ and the positive rate of sputum specimens is higher than that of pharyngeal swab specimens.^24^ In patients with negative SARS-CoV-2 RNA tests in respiratory tract samples, positive tests using anal swabs has been also reported.^25,26^ This may be related to the fact that SARS-CoV-2 enters human cells using the same angiotensin-converting enzyme 2 (ACE2) receptor as severe acute respiratory distress syndrome coronavirus,^5^ and ACE2 mRNA is highly expressed in the human small intestine,^27,28^ so the clearance of virus RNA in feces was delayed after COVID-19 was clinically cured.^29^ Therefore, collecting a variety of samples for virus RNA detection at different times during the disease, especially during the convalescent period, may help reduce the occurrence of a re-detectable positive.

The third possible reason is that the use of antiviral drugs during hospitalization is insufficient. During hospitalization, viral replication was inhibited due to drug treatment, so that the virus load was insufficient or lower than the threshold of the reagent, resulting in negative results. But, in fact, the virus had not been completely cleared. With a lack of treatment and drug reduction after discharge, the proliferation of the virus fluctuated, resulting in the recovery of positive nucleic acid detection. Among the 20 re-detectable positive patients reported by Luo et al.,^21^ 3 cases did not use antiviral therapy, and the average antiviral treatment time of 15 cases was 10.65 days, so it is difficult to explain the phenomenon of a re-detectable positive by the lack of antiviral therapy time.

The fourth possible reason is related to the patient’s course and condition.^30,31^ The amount of virus in the body of patients with different courses and different conditions may be different. COVID-19 has a low viral load and intermittent detoxification at the end of its course,^32,33^ which is manifested by the nucleic acid test being negative during the intermittent period and positive during detoxification. Professor Cao Bin’s team research shows that the median detoxification period of SARS-CoV-2 is 20 days, and the longest detoxification period is 37 days.^34^ However, a COVID-19 patient tested positive for nucleic acid 6 times within 57 days in Italy.^35^

The fifth possible reason is related to the patient’s immune system.^36^ After being infected by SARS-CoV-2, the human body will produce antibodies. Generally speaking, immunoglobulin M (IgM) antibodies are produced about 1 week after infection and last for about half a month. The IgG antibody is produced about half a month after infection and can last for a long time.^37^ Those patients who do not produce antibodies or produce antibodies relatively late will be unable to resist the virus once the immunity decreases, and the number of viruses in the patient’s body will increase again, and the nucleic acid test is also likely to show the re-detectable positive at this time.

**FIGURE 1:**
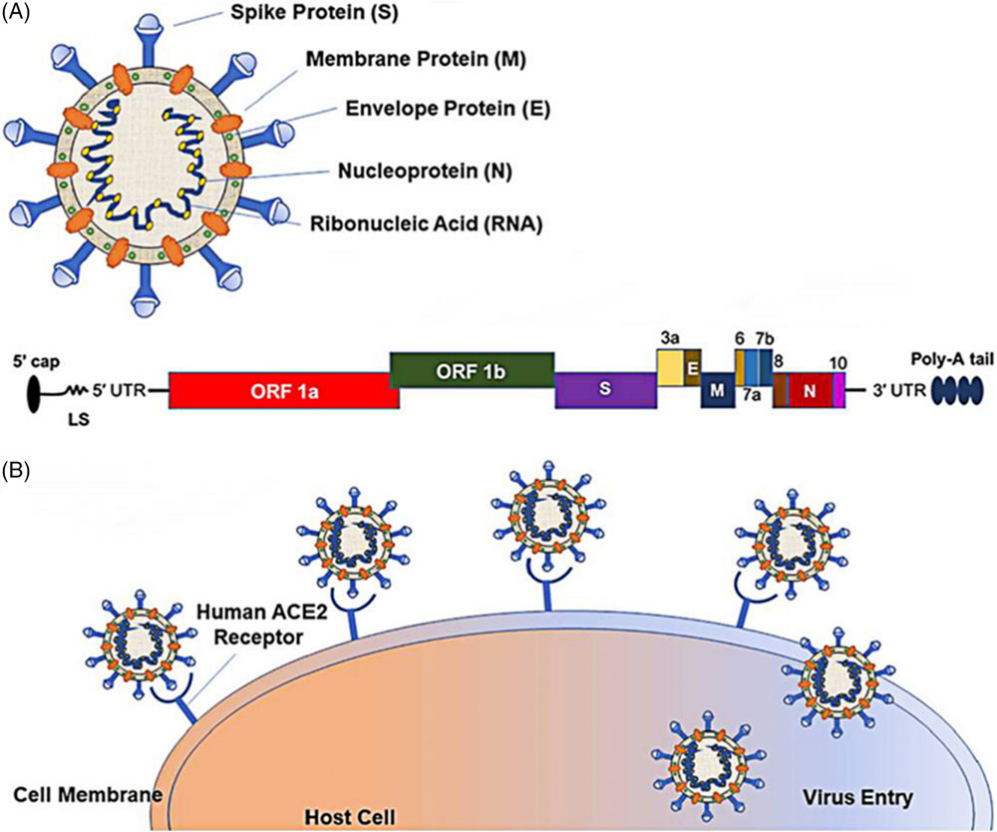
Schematic Representation of the SARS-CoV-2 Structure and Its Mode of Host Entry. *Notes*: (A) Schematic representation of the SARS-CoV-2 structure; 5′ capped mRNA has a leader sequence (LS), poly-A tail at 3′ end, and 5′ and 3′ UTR. It consists of ORF1a, ORF1b, Spike (S), ORF3a, envelope (E), membrane (M), ORF6, ORF7a, ORF7b, ORF8, nucleocapsid (N), and ORF10.^38^ (B) Schematic representation of the SARS-CoV-2 entering the host.

The sixth possible reason is related to the recombination, variation, and continuous passage attenuated virulence of the virus. SARS-CoV-2 is a single-stranded RNA virus, belonging to the β-coronavirus family. It is composed of 4 structural proteins, including spike (S) glycoprotein, membrane (M), envelope (E), and nucleocapsid (N). It also contains the N-terminal domain, the C-terminal domain, and several open reading frames (ORF). The pathogenicity of its infection is closely related to its structural characteristics. The interaction between S glycoprotein and ACE2 receptor to gain entry into cells plays an important role in pathogenesis (Figure 1).^38^ Through the analysis of SARS-CoV-2 variation, it is found that there are many variants of ORF among different strains, which may lead to different immune responses and have different effects on virulence. The re-detectable positive phenomenon may be related to the rapid mutation of SARS-CoV-2 and the natural recombination of the virus,^39^ or it may be the characteristic of this brand-new virus, just because we lack a comprehensive understanding of it. Besides, among the recovered and discharged patients, with the continuous passage of the virus, it may show a trend of weakening toxicity, resulting in the symbiosis of human and virus, thus making COVID-19 patients become asymptomatic carriers, which may lead to a re-detectable positive after discharge.

## HOW TO DEAL WITH THE RE-DETECTABLE POSITIVE PHENOMENON?

For the re-detectable positive patients, serological virus IgM and IgG antibody tests should be added before discharge. Although the nucleic acid test is the “gold standard” for the diagnosis of SARS-CoV-2 infection, it has been reported that there is a certain percentage of false-negative results in nucleic acid testing when patients are discharged from the hospital.^40^ Some researchers believe that the detection of IgG and IgM antibodies for patients previously infected with SARS-CoV-2 can confirm each other with the detection of SARS-CoV-2 RNA, which is helpful to eliminate suspected cases and reduce the risk of missed detection. It has also been reported that asymptomatic carriers need to be diagnosed in combination with serological tests.^41^ Also, all discharged patients should undergo medical observation and quarantine for at least 14 days, and longer periods of observation and surveillance might be necessary. During the isolation period, they should wear a mask, monitor their body temperature, signs, and other physical conditions every day to see whether there is a fever, cough, or other respiratory symptoms.

## CONCLUSION AND FUTURE DIRECTIONS

A lot of information about the epidemiology and clinical medicine of COVID-19 is still unknown. Although there is no evidence of re-detectable positive patients infecting others, it is still necessary to determine whether these re-detectable positive patients will become chronic virus carriers. Further studies on the re-detectable positive patients will be vital for the research and development of a more effective vaccine. It is urgent to find a more powerful evidence-based and virological basis for the integrity of viral RNA and the variation of viral virulence with time through cell experiments in vitro and animal experiments in vivo.
